# Isopeptide and ester bond ubiquitination both regulate degradation of the human dopamine receptor 4

**DOI:** 10.1074/jbc.M116.758961

**Published:** 2017-11-03

**Authors:** Jennifer C. Peeler, Sophia Schedin-Weiss, Mariluz Soula, Manija A. Kazmi, Thomas P. Sakmar

**Affiliations:** From the ‡ Laboratory of Chemical Biology and Signal Transduction, The Rockefeller University, New York, New York 10065 and; § Department of Neurobiology, Care Sciences and Society, Division of Neurogeriatrics, Center for Alzheimer Research, Karolinska Institutet, 141 57 Huddinge, Sweden

**Keywords:** dopamine receptor, G protein-coupled receptor (GPCR), proteasome, protein degradation, ubiquitylation (ubiquitination)

## Abstract

How an optimal level of human dopamine D4 receptor (hD4R) is maintained in synaptic membranes is not known. We show here that hD4R is ubiquitinated in primary neurons. We go on to show that ubiquitin is attached to hD4R through isopeptide and ester bonds. When lysine (Lys) residues of the hD4R are substituted with arginine (Arg) residues, cellular hD4R protein levels increase. A synergistic effect on hD4R levels is noted when cytoplasmic serine (Ser) and threonine (Thr) residues are mutated. Chloroquine, an inhibitor of lysosomal degradation, did not have an effect on hD4R protein levels. However, treatment with bortezomib, an inhibitor of the 20S proteasome, caused a dose-dependent increase in hD4R protein levels. The effect of bortezomib was attenuated in the receptor variants that lacked Lys or Ser/Thr residues, and the hD4R mutant that lacked 17 cytoplasmic Lys, Ser, and Thr residues was nearly insensitive to bortezomib treatment. We conclude that both isopeptide and ester bond ubiquitination regulate proteasomal degradation of hD4R.

## Introduction

The hD4R^3^ is a seven-helical G protein–coupled receptor (GPCR) that binds the modulatory neurotransmitter dopamine. hD4R was discovered through its role as the dopaminergic target of the atypical antipsychotic drug clozapine (1). More recent reports show that neuregulin/ErbB modulation of hippocampal γ oscillations is dependent upon the signaling of D4R but not other dopamine receptors (2, 3). γ oscillations play an important role in cognition, and disrupted γ oscillations are associated with cognitive disorders, including schizophrenia (4). These results have sparked renewed interest in hD4R as a pharmacological target.

hD4R contains a variable number of tandem repeats (VNTR) exon polymorphism in intracellular loop 3 (IC3). A 16-amino-acid, proline (Pro)-rich sequence is repeated two to 11 times in IC3 in human D4R proteins (5). Many studies have found correlations between hD4R tandem repeat number and various aspects of human behavior and mental health. Most correlations have not stood up to meta-analysis, but the association of the seven-repeat variant with attention-deficit/hyperactivity disorder diagnosis is robust (6). It remains unclear whether there is a molecular difference between how variants signal or how they are processed and whether this difference is responsible for the association with attention-deficit/hyperactivity disorder.

Optimal levels of hD4R are required for normal synaptic function, but how hD4R concentration is regulated in the membrane and the potential role of the cytoplasmic tandem repeat variants in the regulation process are not known. We have observed low hD4R protein levels in our protein expression system and noticed highly conserved ubiquitinatable residues on the cytoplasmic surface of the protein. We decided to explore potential ubiquitin-dependent degradation of the receptor. We hypothesize that both canonical, Lys-based isopeptide ubiquitination and non-canonical, Ser- and Thr-based ester bond ubiquitination regulate cellular degradation of hD4R.

## Results

### hD4R is ubiquitinated in transfected primary neurons

**Figure 1.**
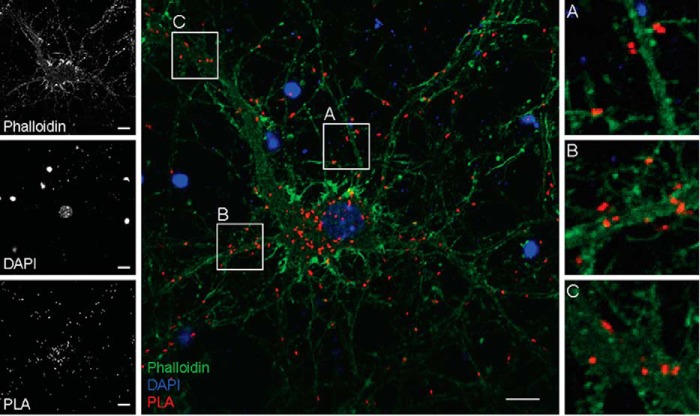
**hD4R ubiquitination in neurons.** PLA analysis shows ubiquitinated WT hD4R-1D4 in transfected primary mouse cortical neurons. Phalloidin (*green*) and DAPI (*blue*) show cellular architecture, whereas the PLA dots (*red*) represent ubiquitinated hD4R. Single channel isolations are shown on the *left*, a full-scale merge is shown in *center*, and on the *right* are zoomed-in views of areas highlighted in the center panel (*A–C*). *Scale bars* represent 10 μm.

To test for hD4R ubiquitination, we utilized the four-repeat hD4R variant, which is the most common variant in the human population (7). We constructed an hD4R synthetic gene with a C-terminal 1D4 mAb epitope (hD4R-1D4) and tested whether hD4R-1D4 is ubiquitinated in transfected primary mouse neurons using proximity ligation assay (PLA) (8). When 1D4 mAb and an Ab against endogenous ubiquitin are in close proximity, a fluorescent dot representative of ubiquitinated hD4R appears (Fig. 1). We detected ubiquitinated hD4R-1D4 in the transfected neurons with some clustering of the ubiquitinated hD4R-1D4 around the nucleus, presumably in the endoplasmic reticulum (ER), and on or near the plasma membrane of both soma and neurites (Fig. 1).

### hD4R mutations separate isopeptide and ester ubiquitination

**Figure 2.**
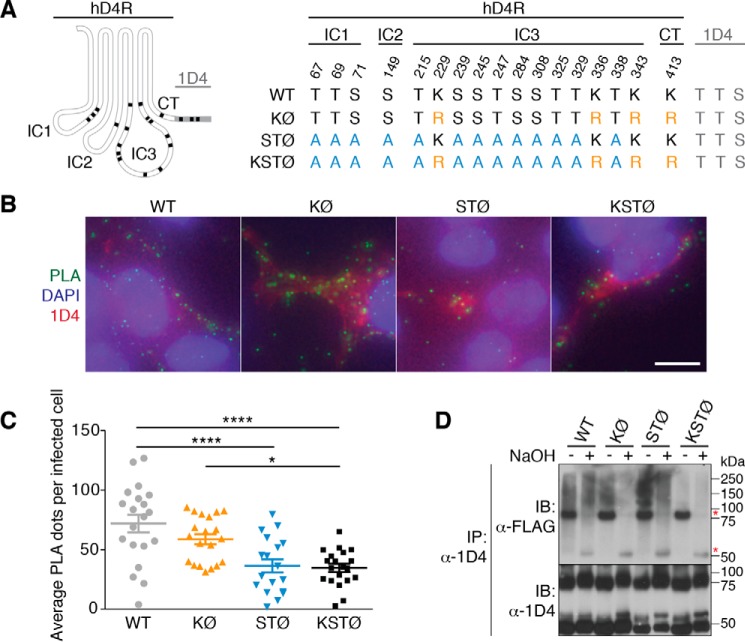
**hD4R mutants show isopeptide and ester ubiquitination.** *A*, schematic of hD4.4R showing 17 potential ubiquitination sites (Lys, Ser, and Thr; *black*). The 1D4 epitope (*gray*) contains three additional potential sites. Three mutants were constructed: KØ, four Lys to Arg (*orange*); STØ, seven Thr to Ala and six Ser to Ala (*blue*); and KSTØ, combined KØ and STØ. Locations of mutated residues are provided according to topology: IC1, IC2, IC3, C terminus (*CT*), or 1D4 epitope. *B*, representative images of PLA on HEK293T infected with baculovirus encoding mutant hD4R-1D4. DAPI (*blue*) shows nuclei, PLA dots (*green*) represent ubiquitinated receptors, and immunofluorescence (*red*) shows cell infection status. *Scale bars* represent 10 μm. *C*, average number of PLA dots per infected HEK293T cell. Five biological replicates were performed per construct, totaling ∼280 infected cells quantified per construct. A one-way ANOVA followed by Tukey's multiple comparison test was used to compare WT and mutant hD4R-1D4. *Error bars* represent S.E. ****, *p* ≤ 0.001; *, *p* ≤ 0.02. *D*, hD4R-1D4 mutants were immunoprecipitated from cell lysates using limiting amounts of 1D4 mAb to achieve equal quantities of each mutant. Eluates were treated ±NaOH before SDS-PAGE and immunoblotting. Immunoblotting (*IB*) with FLAG mAb detects FLAG-ubiquitin. NaOH sensitivity indicates the presence of ester-linked ubiquitination (at Ser/Thr), and NaOH insensitivity indicates isopeptide ubiquitination (at Lys). *Red asterisks* show Ab bands from IP.

We wanted to determine which amino acids in hD4R are modified by ubiquitin attachment. Ubiquitination canonically occurs through isopeptide bonds on Lys residues. There are only four Lys residues in the entire hD4R receptor (1), so we mutated all four Lys residues to Arg to create the KØ hD4R-1D4 mutant (Fig. 2*A*). Recent reports have also indicated that Ser and Thr residues can be ubiquitinated through ester bonds (9–15). Therefore, we also replaced 13 cytoplasmic Ser and Thr residues in hD4R with alanine (Ala) to create STØ hD4R-1D4. Finally, we mutated all Lys and the 13 cytoplasmic Ser and Thr to create KSTØ hD4R-1D4 (Fig. 2*A*).

### hD4R is ubiquitinated through isopeptide and ester bonds

To determine whether wild-type (WT) hD4R-1D4 was being ubiquitinated through isopeptide bonds, ester bonds, or both, we performed PLA on the KØ hD4R-1D4, STØ hD4R-1D4, and KSTØ hD4R-1D4 mutants. HEK293T cells were infected with baculovirus containing WT or mutant hD4R-1D4 cDNA (16). As with the primary neuron PLA, we observed clustering of ubiquitinated WT hD4R-1D4 near the ER and on or near the plasma membrane (Fig. 2*B*). We counted the number of PLA dots for WT hD4R-1D4 and each mutant. There was a statistically significant difference (*p* ≤ 0.001) between the average number of PLA dots per infected cell for WT hD4R-1D4 and the average number for KSTØ hD4R-1D4 (Fig. 2*C*). The difference in PLA dots per cell for WT *versus* KSTØ hD4R-1D4 confirms the specificity of PLA for detecting ubiquitinated receptor. KØ hD4R-1D4 and STØ hD4R-1D4 both had averages less than that of WT hD4R-1D4 but greater than that of KSTØ hD4R-1D4, indicating that isopeptide and ester bond ubiquitination both contribute to WT hD4R-1D4 ubiquitination (Fig. 2*C*). The fact that KØ hD4R-1D4 is statistically most similar to WT hD4R-1D4 and STØ hD4R-1D4 is statistically most similar to KSTØ hD4R-1D4 implies that ester bond ubiquitination may be the predominant form of ubiquitination for hD4R-1D4.

We also tested for hD4R-1D4 ubiquitination with an immunoprecipitation (IP) assay. We carried out IPs using the mutant hD4R-1D4 constructs expressed in HEK293T cells that also expressed FLAG-tagged ubiquitin. We performed the IPs with limiting quantities of the 1D4 mAb to ensure we were comparing equal amounts of mutant hD4R-1D4 protein. In the case of WT hD4R-1D4, there was a high molecular weight FLAG signal indicative of polyubiquitination of the receptor (Fig. 2*D*). We exploited the fact that ester bond ubiquitination is sensitive to base-catalyzed hydrolysis, whereas isopeptide bond ubiquitination is not (11). In the case of WT hD4R-1D4 and the STØ hD4R-1D4 mutant (where Lys residues are present), high molecular weight signal remained after NaOH treatment, indicating that isopeptide ubiquitination was present. In the case of KØ hD4R-1D4 (where Ser and Thr are present but Lys-based ubiquitination is not possible), high molecular weight signal was lost upon base treatment, indicating that the ubiquitin signal was attached to hD4R-1D4 through an ester bond. The KSTØ hD4R-1D4 mutant protein showed little to no high molecular weight FLAG signal regardless of base treatment, consistent with a lack of ubiquitinatable residues (Fig. 2*D*).

Because the PLA and IP experiments indicate the presence of hD4R ubiquitination and a recent study also infers the presence of ester and isopeptide hD4R ubiquitination but does not report cell biological consequences of the modification (17), we wanted to test whether cellular hD4R function and/or protein levels were regulated through ubiquitination.

### hD4R mutants are functional

**Figure 3.**
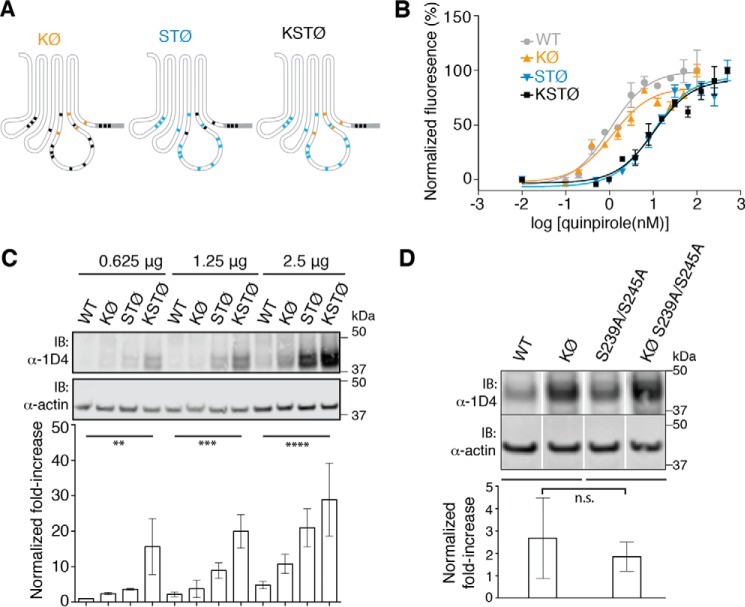
**hD4R mutants are functional and have increased cellular protein levels.** *A*, schematic showing position of Lys to Arg (*orange*) and Ser/Thr to Ala (*blue*) mutations as well as remaining ubiquitinatable residues (*black*). *B*, dose-response curves show extent of calcium release after treatment with the agonist quinpirole for hD4R-1D4 constructs. WT hD4.4R-1D4 EC_50_ is 1.1 nm, KØ EC_50_ is 1.2 nm, STØ EC_50_ is 9.9 nm, and KSTØ EC_50_ is 10.6 nm. Each concentration was assayed in triplicate, and *error bars* represent S.E. Three biological replicates were performed, and a representative dose curve is shown. *C*, protein level of hD4R-1D4 mutants in HEK293T cells transfected with increasing amounts of receptor-specific DNA (0.625, 1.25, and 2.5 μg). The -fold change increases, normalized to actin and compared with WT protein levels at 0.625 μg of DNA, are quantified and graphed. Quantification includes three biological replicates. Significance is shown between WT and KSTØ for each DNA amount. **, *p* ≤ 0.01; ***, *p* ≤ 0.001; ****, *p* ≤ 0.0001 (two-way ANOVA followed by Tukey's multiple comparison test). *D*, lysate protein level of hD4R-1D4 mutants with Ser to Ala mutations at known sites of phosphorylation. HEK293T cells were transfected with 0.875 μg of DNA. *White lines* show locations of image splicing to remove irrelevant lanes. The -fold change increase of KØ protein levels over WT protein levels (normalized to actin) and the -fold change increase of KØ S239A/S245A protein levels over S239A/S245A protein levels (normalized to actin) are graphed. Quantification includes three biological replicates. There is no statistically significant difference (*n.s.*) according to Student's *t* test when comparing the -fold change of KØ over WT with the -fold change of KØ S239A/S245A over S239A/S245A. This result suggests that Ser phosphorylation at these sites does not influence the extent of hD4R Lys-dependent changes in protein levels. *IB*, immunoblotting.

Mutants (Fig. 3*A*) were tested for function in an agonist-dependent (quinpirole) intracellular calcium release assay. The EC_50_ for the effect of quinpirole on KØ hD4R-1D4 was unchanged from that of WT hD4R-1D4. Both STØ hD4R-1D4 and KSTØ hD4R-1D4 remained functional, although they showed EC_50_ values that were shifted about 10-fold as compared with the EC_50_ for WT (Fig. 3*B*). These relatively modest differences in EC_50_ values can likely be explained by the influence of cytoplasmic substitutions on G protein coupling or the effect of G protein precoupling on agonist affinity or a potential difference in hD4R protein concentration in the plasma membrane between mutants (18, 19).

### hD4R mutants have different steady-state protein levels

Recent reports have shown that both isopeptide bond ubiquitination at Lys residues and ester bond ubiquitination at Ser and Thr residues can serve to regulate protein abundance (9, 10). To test whether isopeptide and/or ester bond ubiquitination regulates hD4R protein levels, we measured steady-state receptor levels in cell lysates of transfected HEK293T cells. The protein level of KØ hD4R-1D4 was slightly greater than that of WT hD4R-1D4. We also found that STØ hD4R-1D4 protein levels were increased compared with WT hD4R-1D4. KSTØ hD4R-1D4 protein levels were greatly increased compared with WT hD4R-1D4, KØ hD4R-1D4, and STØ hD4R-1D4, showing an additive influence of Lys, Ser, and Thr residues (Fig. 3*C*). This trend held true when we transfected increasing amounts of receptor-specific plasmid. In fact, cells transfected with 0.625 μg of DNA encoding KSTØ had hD4R-1D4 protein levels 3 times greater than the hD4R-1D4 protein levels in cells transfected with 2.5 μg of DNA encoding WT receptor. These data support the hypothesis that both isopeptide bond and ester bond ubiquitination regulate hD4R protein levels post-translationally.

### hD4R phosphorylation is not required for isopeptide ubiquitination

Phosphorylation has been detected through mass spectrometry on Ser-239 and Ser-245, although no physiological role has been uncovered for these modifications (20, 21). Cross-talk between Ser and Thr phosphorylation and Lys ubiquitination has been described previously, including the phosphorylation-dependent binding of the E3 ubiquitin ligase AIP4 to the chemokine receptor CXCR4 (22). To investigate whether Ser-239 and Ser-245 phosphorylation is required for the isopeptide ubiquitin-dependent increase in hD4R protein level, we created two additional mutants: S239A/S245A hD4R-1D4 where all Lys residues are present and KØ S239A/S245A hD4R-1D4 where all ubiquitinatable Lys residues are mutated to Arg. If phosphorylation of Ser-239 and Ser-245 was required for ubiquitination of hD4R at Lys residues, then protein levels of S239A/S245A hD4R-1D4 and KØ S239A/S245A hD4R-1D4 should be equal. In fact, the protein level of KØ S239A/S245A hD4R-1D4 was greater than that of S239A/S245A hD4R-1D4 (Fig. 3*D*). The protein level difference between KØ S239A/S245A hD4R-1D4 and S239A/S245A hD4R-1D4 was comparable with the difference in protein level between KØ hD4R-1D4 and WT hD4R-1D4 (Fig. 3*D*), indicating that isopeptide ubiquitin-dependent changes in hD4R protein levels do not require hD4R phosphorylation. The fact that S239A/S245A hD4R-1D4 protein levels are greater than WT hD4R-1D4 protein levels suggests that Ser-239 and/or Ser-245 may be subject to ester bond ubiquitination, which regulates in part the hD4R protein level. In other words, substitution of these two Ser residues removes potential sites of ubiquitination that may be promoting degradation of WT hD4R.

### hD4R mutation increases protein stability

**Figure 4.**
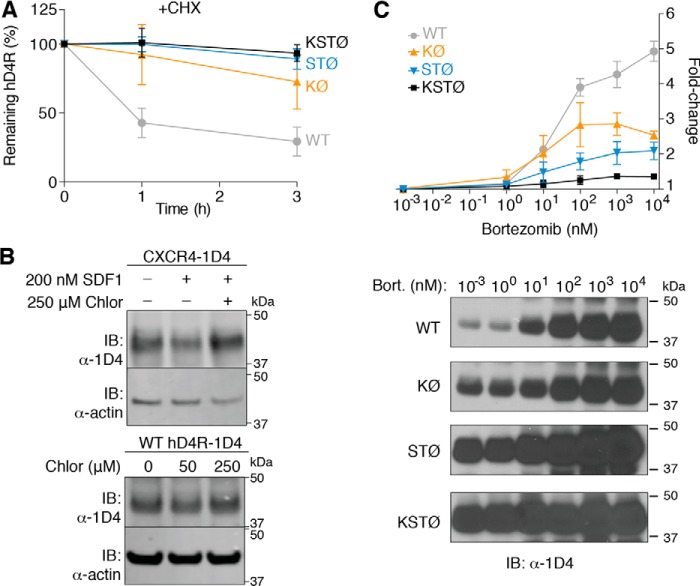
**hD4R proteasomal degradation.** *A*, protein stability of hD4R-1D4 mutants as measured by immunoblotting (*IB*) in the presence of the protein translation inhibitor cycloheximide (*CHX*; 50 μm/ml). Three biological replicates were performed, and *error bars* represent S.E. *B*, HEK293T cells expressing CXCR4–1D4 were either untreated, treated with SDF1α to induce lysosomal degradation, or treated with SDF1α and the lysosomal inhibitor chloroquine diphosphate (*Chlor*) (*top panel*). HEK293T cells expressing WT hD4R-1D4 were incubated with increasing concentrations of chloroquine diphosphate (*bottom panel*). *C*, relative -fold change in lysate protein level, as measured by immunoblotting, for hD4R-1D4 mutants over increasing concentrations of the proteasome inhibitor bortezomib (*Bort.*). At least two biological replicates were performed, and *error bars* represent S.E. Representative blots for each mutant are shown.

The increase in hD4R-1D4 protein levels upon mutation of ubiquitinatable Lys, Ser, and Thr residues led us to hypothesize that WT hD4R-1D4 protein is being degraded as a result of ubiquitination. To test the hypothesis that WT hD4R is degraded, we used cycloheximide to inhibit protein translation for 0–3 h in HEK293T cells expressing WT or mutant hD4R-1D4 (Fig. 4*A*). The protein levels for WT hD4R were reduced by about one-half after 1 h of incubation with cycloheximide. The KØ, STØ, and KSTØ hD4R-1D4 mutant proteins were all more stable than WT hD4R-1D4 with half-lives greater than 3 h. The increase in protein stability upon mutation of Lys, Ser, and Thr residues supports the hypothesis that WT hD4R protein is degraded in a ubiquitin-dependent manner.

### hD4R is degraded proteasomally through isopeptide and ester bond ubiquitination

Screens with small molecule inhibitors that target various protein degradation pathways were carried out to evaluate how WT hD4R-1D4 was being degraded. Ubiquitinated GPCRs are canonically down-regulated by lysosomal degradation. However, chloroquine, an inhibitor of lysosomal degradation, did not have an effect on hD4R protein levels. A dose of 250 μm chloroquine was sufficient to inhibit the SDF1α-induced lysosomal degradation of the chemokine receptor CXCR4–1D4 but did not increase hD4R-1D4 protein levels (Fig. 4*B*) (23). We found that treatment of cells expressing hD4R-1D4 with bortezomib, an inhibitor of the 20S subunit of the proteasome, resulted in large increases in hD4R-1D4 protein levels (24). There was a positive dose-response relationship between bortezomib concentration and WT hD4R-1D4 protein level beginning at 10 nm bortezomib (Fig. 4*C*). These results show that WT hD4R-1D4 is degraded proteasomally and is sensitive to bortezomib treatment.

A dose-response curve was then measured with bortezomib and KØ hD4R-1D4. There was also a dose-dependent increase in the level of KØ hD4R-1D4 with increasing concentrations of bortezomib. The total extent of the increase for KØ hD4R-1D4 was about half that of WT hD4R-1D4. STØ hD4R-1D4 displayed a dose-dependent increase in protein level as well, also totaling about half of that displayed by WT hD4R-1D4. The KSTØ hD4R-1D4 mutant showed very little increase in protein level with increasing concentrations of bortezomib, suggesting that the vast majority of hD4R-1D4 proteasomal degradation was dependent on Lys, Ser, and Thr residues in combination and not on other forms of ubiquitination such as thioester (Fig. 4*C*). These results support the hypothesis that isopeptide and ester bond ubiquitination both contribute to degradation of the WT hD4R receptor.

## Discussion

According to the canonical ubiquitination pathway, ligand-activated GPCRs at the plasma membrane are ubiquitinated and internalized, which promote their lysosomal degradation over recycling back to the cell surface (23). Our studies of how cells control levels of the hD4R through ubiquitination suggest a deviation from the canonical GPCR ubiquitination model in two ways. First, using both PLA in primary cultured neurons and PLA and IP in cultured cell lines, we detected ubiquitination of hD4R in the absence of exogenous agonist ligands (Figs. 1 and 2, *B*, *C*, and *D*). Second, treatments with lysosomal and proteasomal inhibitors (Fig. 4, *B* and *C*) demonstrate that ubiquitin-dependent degradation of hD4R is proteasomal. Taken together, our data support a model of extensive proteasomal degradation of hD4R following ligand-independent ubiquitin.

Our results may be more consistent with a role of ubiquitination and proteasomal degradation of misfolded GPCRs in the ER as is the case for the δ-opioid receptor (25). PLA detection of the subcellular localization of ubiquitinated receptor does demonstrate that a perinuclear population of ubiquitinated hD4R exists. It is possible that the perinuclear population is ubiquitinated in the ER due to misfolding and subsequently proteasomally degraded. However, in primary mouse cortical neurons as well as HEK293T cells, ubiquitinated receptors were also detected at the plasma membrane. As shown by the chloroquine dose curve experiments (Fig. 4*B*), these plasma membrane receptors are not being lysosomally degraded after ubiquitination. The consequence of ubiquitination of plasma membrane hD4R is not clear but may be an example of proteasomal degradation of plasma membrane proteins (as has previously been observed for opioid receptors (26)) or a currently unappreciated role for ubiquitination in hD4R signaling.

In addition, we have detected a role for ester bond ubiquitination in hD4R degradation. Non-isopeptide (non-Lys and non-N-terminal) ubiquitination was first discovered in 2005 when Cadwell and Coscoy (9) reported ubiquitination of a “Lys-less” MHC-I cytoplasmic domain through a viral E3 ligase. More recent examples show that Lys-less proteins can be ubiquitinated in mammalian, insect, plant, and yeast cells regardless of viral infection (10, 12, 27, 28). We have demonstrated that the Lys-less KØ hD4R-1D4 mutant is ubiquitinated and proteasomally degraded (Figs. 2, *C* and *D*, and 4*C*). Additionally, we have characterized a cytoplasmically Ser-less and Thr-less mutant, STØ hD4R-1D4 (Figs. 2*A* and 3*A*), that still retains protein function (Fig. 3*B*). We have confirmed, not just inferred, the identity of the non-Lys ubiquitination through quantifying the ubiquitination and proteasomal degradation of STØ hD4R-1D4 (Figs. 2*C* and 4*C*). Finally, we have made a functional Lys-less and cytoplasmically Ser-less and Thr-less mutant, KSTØ hD4R-1D4 (Figs. 2*A* and 3*A*), where ubiquitination and proteasomal degradation are substantially diminished and the protein half-life is substantially increased compared with WT hD4R-1D4 (Figs. 2*C*, and 4, *A* and *C*).

Before an ubiquitinated GPCR at the plasma membrane can be lysosomally degraded, it must be internalized. GPCR desensitization via internalization is canonically dependent upon agonist-induced phosphorylation of cytoplasmic Ser and Thr residues via G protein–coupled receptor kinases. Ser and Thr phosphorylation would be temporally mutually exclusive with Ser and Thr ubiquitination. Intriguingly, hD4R has been previously shown to be resistant to agonist-induced desensitization via the canonical pathway (20). The same study qualitatively detected basal phosphorylation at residues Ser-239 and Ser-245 but found no physiological role for the modification. Phosphorylation at Ser-239 and Ser-245 is not ligand-dependent and is not responsible for β-arrestin recruitment to hD4R (20, 21). Some studies have found a role for GPCR phosphorylation in promoting ubiquitination. For example, CXCR4 is ubiquitinated on Lys residues by the E3 ligase AIP4, which binds the receptor in a phosphorylation-dependent manner (22). We determined that Ser-239 and Ser-245 are not required for hD4R Lys-dependent degradation, suggesting that phosphorylation at these sites is not required for isopeptide ubiquitination of hD4R (Fig. 3*D*).

It was previously observed by Van Craenenbroeck and co-workers (29) that hD4R is ubiquitinated. They discovered an interaction between hD4R and the ubiquitin ligase adaptor KLHL12 through a yeast two-hybrid screen. They have since studied the KLHL12-dependent ubiquitination of hD4R through overexpression of the ligase adaptor. They concluded that overexpression of KLHL12 induced ubiquitination of hD4R on Lys, Ser, and Thr but did not influence hD4R protein levels (17, 29). We have in fact observed substantial influence of ubiquitination on hD4R protein levels. It may be that degradation-inducing ubiquitination described here is separate from the previously described KLHL12-dependent ubiquitination, or it may be that, because the earlier work only monitored for degradation of hD4R under KLHL12 overexpression conditions, the rate of degradation due to endogenous KLHL12 in HEK cells was not fully appreciated.

The hD4R contains a VNTR in IC3. It is important to note that the 16-amino-acid repeat sequence is not perfectly replicated in each repeat region. Although all repeat segments are Pro-rich, the amino acid identity between Pro residues is variable both within a given allele and among the alleles present in the human population. Intriguingly, despite the substantial differences in total amino acid number among hD4R variants, the ubiquitinatable residues (Lys, Ser, and Thr) are nearly constant for the most common two-repeat, four-repeat, and seven-repeat alleles (7). We hypothesize, therefore, that ubiquitin-based degradation of hD4R is consistent across common variants. Some rare variants exist at low abundance in the human population that have greater numbers of ubiquitinatable residues (5), and the protein levels of these variants should be investigated further.

Both presynaptic and postsynaptic activation of D4R in the brain have numerous physiological consequences (30). For example, a role is emerging for D4R in regulation of γ oscillation power, highlighting the specific importance of D4R in cognition compared with other dopamine receptors (2, 3). These types of observations highlight the importance of cellular mechanisms to control the levels of cell surface expression of hD4R. In summary, our work supports the hypothesis that proteasomal degradation through both isopeptide and ester bond ubiquitination regulate hD4R levels.

## Experimental procedures

### Plasmids

The WT pcDNA3.1-hD4R-1D4 construct was described previously (31). Mutations were made utilizing the QuikChangeLightning Multi Site-Directed Mutagenesis kit (Agilent Technologies). Mutants were added to pFBDM using EcoRI/NotI restriction enzyme sites. pCMV10–3XFLAG-ubiquitin was a gift from Andrian Marchese, Medical College of Wisconsin, and qi5 was a gift from Bruce Conklin (Addgene plasmid 24501).

### Immunoblotting

HEK293T cells were transiently transfected with Lipofectamine and Plus reagents and grown for 48 h. For transfections with increasing amounts of receptor-specific plasmid, the total DNA level in each transfection was kept the same by supplementing with empty pcDNA3.1 plasmid. After harvesting, cell pellets were solubilized with radioimmune precipitation assay buffer with protease inhibitors, PMSF, NaF, Na_3_VO_4_, and Benzonase at 4 °C for 1 h. Lysates were spun at 10,000 rpm at 4 °C for 10 min and then normalized for total protein using the DC Protein Assay (Bio-Rad). Lysates were analyzed by SDS-PAGE (using a 4–12% Bis-Tris gel) followed by immunoblotting on PVDF membrane with 1D4 Ab (32) and anti-mouse HRP secondary antibody (Novex 16066). A sample processing control was analyzed by SDS-PAGE and immunoblotting with actin Ab (Abcam 8227) and anti-rabbit HRP secondary antibody (Novex 16110). In the case of DNA titration experiments, chloroquine experiments, phosphorylation mutant experiments, and cycloheximide experiments, IRDye-conjugated secondary Abs were used for 1D4 detection and the actin loading control, and blots were imaged on a LI-COR Biosciences Odyssey. For lysosome inhibition experiments, cells were grown in the presence of 50 or 250 μm chloroquine diphosphate (Sigma) for the final 4 h and 200 nm SDF1α (PeproTech) for the final 3.5 h before harvesting. For proteasomal inhibition experiments, cells were grown in the presence of bortezomib (Cell Signaling Technology; 1 pm to 1 mm) for the final 12 h before harvesting. For protein stability experiments, cells were incubated with 50 μg/ml cycloheximide (Cell Signaling Technology) for the final 3 or 1 h before harvesting, or cycloheximide was added immediately before harvest (time = 0 h).

### Immunopurification

HEK293T cells were transfected as above with pcDNA3.1-hD4R-1D4 constructs and 3XFLAG-ubiquitin. After lysing and normalizing as above, cell lysates were incubated with limiting quantities of 1D4 Ab-coated protein G Dynabeads (Invitrogen 10003D) and incubated overnight. Beads were washed, and then the protein was specifically eluted with 1D5 peptide. Elutions were treated with peptide:*N*-glycosidase F (New England Biolabs) and then split in half. One aliquot was incubated with 50 mm NaOH (pH 12.3) for 1 h at 37 °C. The other aliquot was incubated with an equivalent volume of PBS (pH 7.6) for 1 h at 37 °C. Samples were then analyzed by SDS-PAGE and immunoblotting as before with both 1D4 and anti-FLAG-M2 Abs (Sigma-Aldrich) and anti-mouse HRP secondary Ab.

### Calcium flux assay

HEK293T cells were cotransfected with pcDNA3.1-hD4.4R-1D4 and qi5 using Lipofectamine 2000. Cells were transfected directly into a poly-d-lysine–coated 384-well plate. After 48 h, cells were incubated with FLIPR Calcium 4 Assay kit dye for 1 h in the tissue culture incubator. The fluorescence (excitation, 488 nm; emission, 530 nm) of the wells was then recorded using Flex Station II 384 (Molecular Devices) while a dose curve of quinpirole was added to wells, in triplicate, from 0 to 100 nm (WT and KØ) or 0 to 500 nm (STØ and KSTØ). Normalized maximum-minimum fluorescence for each well was plotted for each dose curve, and a four-parameter fit was used to calculate EC_50_ for each construct. Dose curves were repeated three times on independently prepared samples, and a representative dose curve is shown.

### Culturing of primary neurons

Cortices were dissected from C57Bl/6 16–17-day (E16.5) mouse embryos essentially as described previously (33, 34). The isolated cortex neurons were seeded on the inner well of 35-mm glass-bottom dishes Number 1.5 (MatTek Corp.) that had been pretreated with poly-d-lysine. The neurons were grown for 6 days *in vitro* in selective Neurobasal medium containing 2% B27 (Invitrogen) and 1% l-glutamine (Invitrogen) at 37 °C in a cell incubator (humidified; 5% CO_2_) until the day of transfection.

### PLA

Primary neurons were transfected with 0.2 μg of pcDNA3.1-hD4R-1D4 using 0.6 μl of Lipofectamine 3000 and 0.4 μl of P3000. Growth medium was removed to keep a total volume of 70 μl during the initial stage of the transfection. After 4-h incubation, an excess of growth medium was re-added to the dishes. After 48 h, cells were washed twice in DPBS, fixed in 10% formalin for 10 min at room temperature, washed again with DPBS, and permeabilized with 0.4% CHAPSO for 10 min at room temperature. Cells were then processed following the manufacturer's instructions for DuoLink Detection Reagents Far Red (Sigma-Aldrich) using anti-1D4 and anti-ubiquitin (Abcam 7780) primary Abs. After PLA processing, cells were stained with phalloidin-Alexa Fluor 488 to visualize neuronal architecture. Samples were mounted in DuoLink mounting medium with DAPI (Sigma-Aldrich).

For quantitative PLA, HEK293T cells were infected with pFBDM baculovirus prepared as described previously (16). Cells were plated on 35-mm glass-bottom dishes Number 1.5 in the presence of 800 μl of virus-containing medium. Plates were incubated for 48 h before processing. Cells were washed once in DPBS, fixed in cold methanol for 5 min at −20 °C, and washed three more times with DPBS. PLA was performed as written for neurons but using the Abcam 134953 ubiquitin primary Ab and the DuoLink Detection Reagents Green (Sigma-Aldrich) kit. Anti-mouse Alexa Fluor 594 fluorescent secondary Ab was used to detect infected cells.

### Image acquisition

Confocal images of PLA-treated neurons were acquired with a Nikon A1RSi point scanning confocal inverted microscope using a 60× oil immersion objective. Excitation lasers of 405, 488, and 640 nm were used. Images from single confocal planes are shown. Image processing (cropping and adding of scale bars) was done in NIS Elements. The brightness and intensity of the images were adjusted in Adobe Photoshop CS6 to enhance the possibility to observe fine structures on printouts, and the final figure was made in Adobe Illustrator CS6.

For quantitative PLA in HEK293T cells, 20× magnification Z-stack images (every 1.5–2 μm) were captured using an FSX100 Olympus microscope. Exposure times were held constant while imaging samples within each experimental replicate. A total of four fields of view were obtained per sample per experiment.

### Data analysis of quantitative PLA

All image processing was done using ImageJ. Nuclei stained with DAPI were counted to obtain the total number of cells per image. Then the number of infected cells, identified as nuclei surrounded by a full ring of 1D4 immunofluorescence, were counted. The total number of PLA dots per field was determined by creating a maximum projection of the PLA channel, converting it to a 32-bit grayscale image, and recording the number of dots as the number of PLA signals.

The average number of PLA signals per cell of a control-infected plate was used to estimate background signal for each experiment. For each field to be quantified, the average number of background PLA dots per cell (averaged over the four images obtained from the control infection) was multiplied by the total number of cells in that field. This number was then subtracted from the total number of PLA dots in that field to remove background. The remaining dots were divided by the number of infected cells to account for variable infection efficiencies, resulting in the average number of PLA dots per infected cell per field. For each of the five biological replicates there were four fields per sample, resulting in a total of 20 data points per sample. Using Prism, a one-way ANOVA followed by Tukey's multiple comparison test was used to compare the mean of each group with that of every other group. Significance was determined by a *p* value <0.05.

## Author contributions

J. C. P. and T. P. S. conceived the overall project. J. C. P. designed and performed HEK293T experiments. M. S. and J. C. P. designed and performed quantitative PLA in HEK293T cells. S. S.-W. and J. C. P. designed and performed mouse neuron experiments. M. A. K. performed cycloheximide and DNA titration experiments. T. P. S. and J. C. P. wrote the manuscript.

## Footnotes

3 The abbreviations used are:

hD4R: human dopamine D4 receptor
GPCR: G protein–coupled receptor
VNTR: variable number of tandem repeats
IC: intracellular loop
PLA: proximity ligation assay
Ab: antibody
ER: endoplasmic reticulum
IP: immunoprecipitation
Bis-Tris: 2-[bis(2-hydroxyethyl)amino]-2-(hydroxymethyl)propane-1,3-diol
DPBS: Dulbecco's PBS
CHAPSO: 3-[(3-cholamidopropyl)dimethylammonio]-2-hydroxy-1-propanesulfonic acid
ANOVA: analysis of variance.
